# Supplementary material for: Effect direction meta-analysis of GWAS identifies extreme, prevalent and shared pleiotropy in a large mammal
Source: Commun Biol. 2020 Feb 28;3:88. doi: 10.1038/s42003-020-0823-6 (PMC7048789; doi:10.1038/s42003-020-0823-6)
Supplement: Supplementary file 4 — Description of Additional Supplementary Files [file 42003_2020_823_MOESM4_ESM.pdf]

Items in the Supplementary Data in excel form:

Supplementary Data 1. Trait characteristics for bulls and cows.

Supplementary Data 2. The overlap of the number of significant variants (bold numbers) detected in different meta-analyses.

Supplementary Data 3. Conventional false discovery rate for meta-analysis p values in bulls, in cows and in the combination of bulls and cows.

Supplementary Data 4. Features of variants with consistent effect directions, false discovery rate by effect direction (FDRed) and the conventional FDR at different single-trait p value thresholds averaged across 34 traits.

Supplementary Data 5. Characteristics of false discovery rates (FDR) and predicted True Effects (TE) on traits at thresholds of p values for multi-trait meta-analysis in both sexes.

Supplementary Data 6. Top variants within each category of pleiotropy based on the number of traits the variant truly affected (column trn).

Supplementary Data 7. A set of variants with unevenly distributed pleiotropic effects.

Supplementary Data 8. top 1000 variants ranked by Pi value with pleiotropic effects not led by production traits.

Supplementary Data 9. Summary of results of Mendelian Randomisation (MR) for 50 pairs of raw traits prioritised by variant sharing after Cholesky transformation.

Supplementary Data 10. Pruned novel pleiotropic variants identified in the current study compared to the 2017 study (Xiang et al 2017).

Supplementary Data 11. summary statistics for 37 original traits (not Cholesky decorrelated).

Supplementary Data 12. Details of original traits selected for validation using gBLUP to predict 1000-bull individuals.
