# Supplementary material for: Effect direction meta-analysis of GWAS identifies extreme, prevalent and shared pleiotropy in a large mammal
Source: Commun Biol. 2020 Feb 28;3:88. doi: 10.1038/s42003-020-0823-6 (PMC7048789; doi:10.1038/s42003-020-0823-6)
Supplement: Supplementary file 5 — Supplementary Information [file 42003_2020_823_MOESM5_ESM.pdf]

A

Order	trait$_{ct}$	traits corrected for																																
		1.Prot	2.ProtP	3.FatP	4.Milk	5.SCC	6.Fert	7.SurvDi	8.Temp	9.MSpeed	10.Like	11.CentL	12.PinW	13.PinSet	14.RSet	15.ForeA	16.RearAH	17.RearAW	18.TeatPF	19.Stat	20.Angul	21.Bone	22.ChestW	23.MuzW	24.UdTex	25.OType	26.Mamm	27.BodyD	28.FootA	29.TeatL	30.UdDep	31.Loin	32.RLeg	33.TeatPR
1	Prot																																	
2	ProtP	1																																
3	FatP	1	2																															
4	Milk	1	2	3																														
5	SCC	1	2	3	4																													
6	Fert	1	2	3	4	5																												
7	SurvDi	1	2	3	4	5	6																											
8	Temp	1	2	3	4	5	6	7																										
9	MSpeed	1	2	3	4	5	6	7	8																									
10	Like	1	2	3	4	5	6	7	8	9																								
11	CentL	1	2	3	4	5	6	7	8	9	10																							
12	PinW	1	2	3	4	5	6	7	8	9	10	11																						
13	PinSet	1	2	3	4	5	6	7	8	9	10	11	12																					
14	RSet	1	2	3	4	5	6	7	8	9	10	11	12	13																				
15	ForeA	1	2	3	4	5	6	7	8	9	10	11	12	13	14																			
16	RearAH	1	2	3	4	5	6	7	8	9	10	11	12	13	14	15																		
17	RearAW	1	2	3	4	5	6	7	8	9	10	11	12	13	14	15	16																	
18	TeatPF	1	2	3	4	5	6	7	8	9	10	11	12	13	14	15	16	17																
19	Stat	1	2	3	4	5	6	7	8	9	10	11	12	13	14	15	16	17	18															
20	Angul	1	2	3	4	5	6	7	8	9	10	11	12	13	14	15	16	17	18	19														
21	Bone	1	2	3	4	5	6	7	8	9	10	11	12	13	14	15	16	17	18	19	20													
22	ChestW	1	2	3	4	5	6	7	8	9	10	11	12	13	14	15	16	17	18	19	20	21												
23	MuzW	1	2	3	4	5	6	7	8	9	10	11	12	13	14	15	16	17	18	19	20	21	22											
24	UdTex	1	2	3	4	5	6	7	8	9	10	11	12	13	14	15	16	17	18	19	20	21	22	23										
25	OType	1	2	3	4	5	6	7	8	9	10	11	12	13	14	15	16	17	18	19	20	21	22	23	24									
26	Mamm	1	2	3	4	5	6	7	8	9	10	11	12	13	14	15	16	17	18	19	20	21	22	23	24	25								
27	BodyD	1	2	3	4	5	6	7	8	9	10	11	12	13	14	15	16	17	18	19	20	21	22	23	24	25	26							
28	FootA	1	2	3	4	5	6	7	8	9	10	11	12	13	14	15	16	17	18	19	20	21	22	23	24	25	26	27						
29	TeatL	1	2	3	4	5	6	7	8	9	10	11	12	13	14	15	16	17	18	19	20	21	22	23	24	25	26	27	28					
30	UdDep	1	2	3	4	5	6	7	8	9	10	11	12	13	14	15	16	17	18	19	20	21	22	23	24	25	26	27	28	29				
31	Loin	1	2	3	4	5	6	7	8	9	10	11	12	13	14	15	16	17	18	19	20	21	22	23	24	25	26	27	28	29	30			
32	RLeg	1	2	3	4	5	6	7	8	9	10	11	12	13	14	15	16	17	18	19	20	21	22	23	24	25	26	27	28	29	30	31		
33	TeatPR	1	2	3	4	5	6	7	8	9	10	11	12	13	14	15	16	17	18	19	20	21	22	23	24	25	26	27	28	29	30	31	32	
34	BCS	1	2	3	4	5	6	7	8	9	10	11	12	13	14	15	16	17	18	19	20	21	22	23	24	25	26	27	28	29	30	31	32	

B

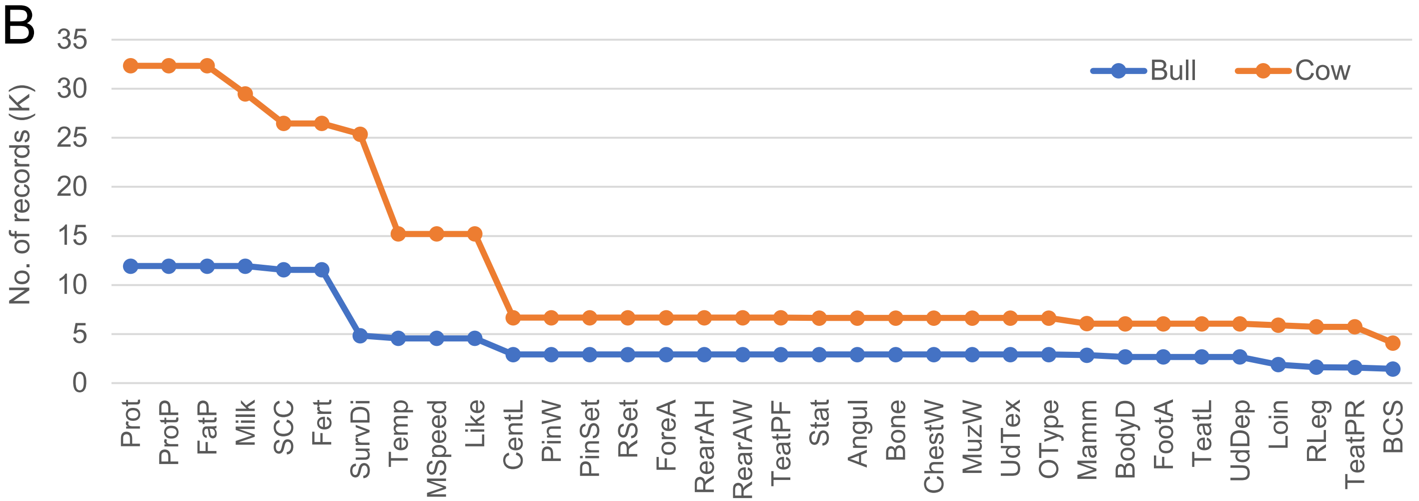

Supplementary Figure 1. Details of the Cholesky transformed (CT) traits. **A**: The interpretation of the Cholesky transformed traits (trait$_{ct}$). Each CT trait is interpreted as the trait corrected by all of its preceding traits. The 1st column of the table is the order of Cholesky transformation. The 2nd column is the CT trait name. From the 3rd column, raw traits that were corrected for the CT trait are specified. The number in cells indicate the traits corrected based on its order in the transformation. For example, the 1st CT Prot had no prior traits to be corrected for so it is identical to the raw Prot. The 2nd CT trait ProtP is interpreted as corrected for its preceding trait Prot (1.Prot). The 3rd CT trait FatP is interpreted as corrected for its preceding traits including the 1st trait Prot (1) and the 2nd trait ProtP (2.ProtP). Such interpretation goes on until the last CT trait BCS. **B**: The order of the Cholesky transformation is determined by the completeness of trait records in both bulls and cow populations. Within each population, due to a varying number records for each trait, we ordered the traits from the largest number of records (e.g., Prot) to the smallest number of records (e.g., BCS). Moreover, our study requires decorrelated traits matched in both populations to make sure that the GWAS of the same trait was carried out in two populations. Therefore, the trait order of Cholesky transformation needs to suit the trait completeness in both bulls and cows.

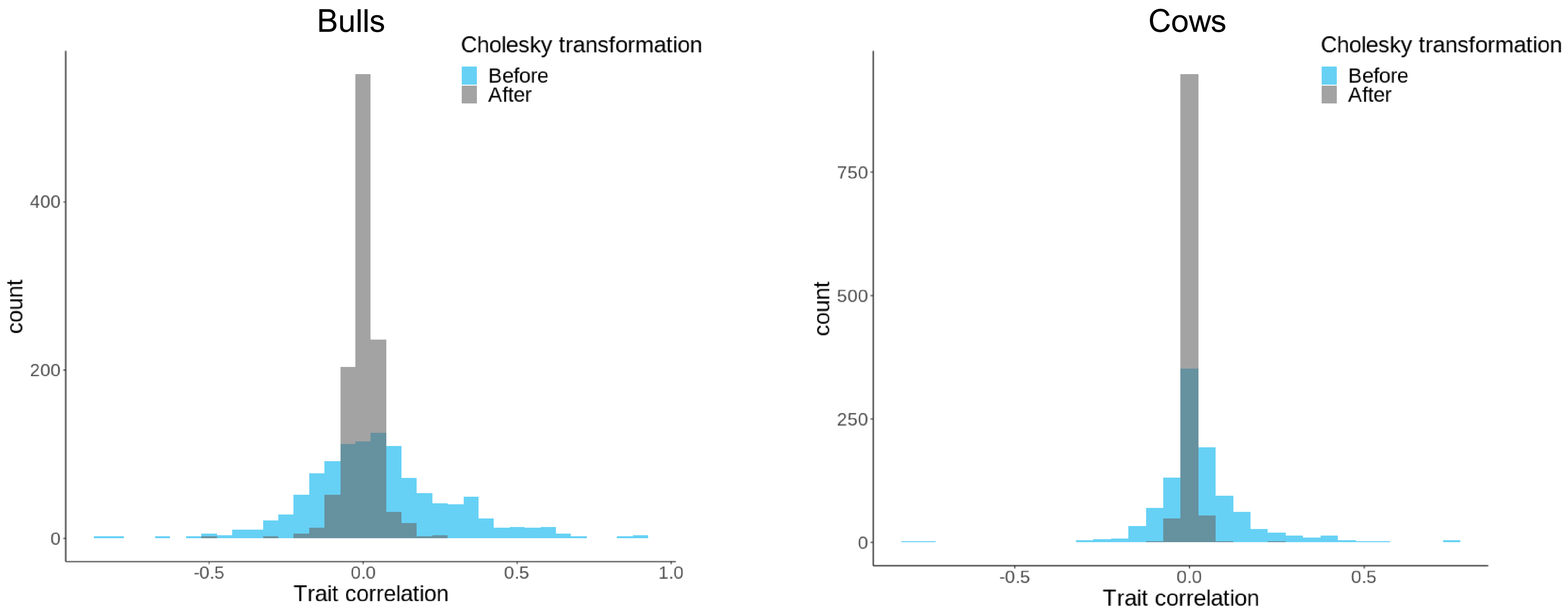

Supplementary Figure 2. The distribution of the pairwise trait correlations before and after Cholesky transformation in both sexes.

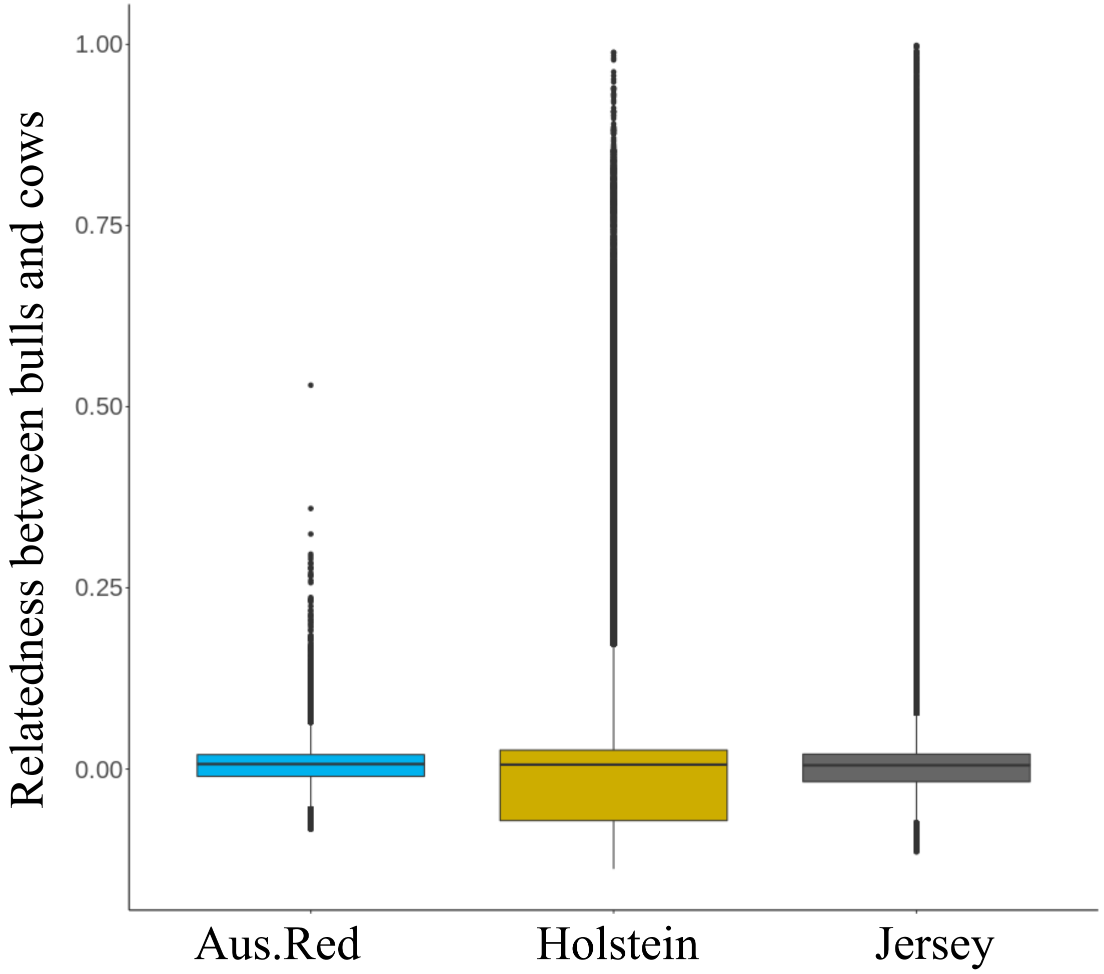

Supplementary Figure 3. The boxplot of distribution of the genomic relationships between bull and cow populations for breeds of Australian Red (Aus.Red), Holstein and Jersey. The genomic relationships (across-sex off-diagonal elements) were extracted from the genomic relationship matrix made by GCTA [1] with 16M+ sequence variants with MAF >0.001 in 44,000+ bulls and cows.

[1]: Yang J, Lee SH, Goddard ME, & Visscher PM (2011) GCTA: a tool for genome-wide complex trait analysis. *The American Journal of Human Genetics* 88(1):76-82.

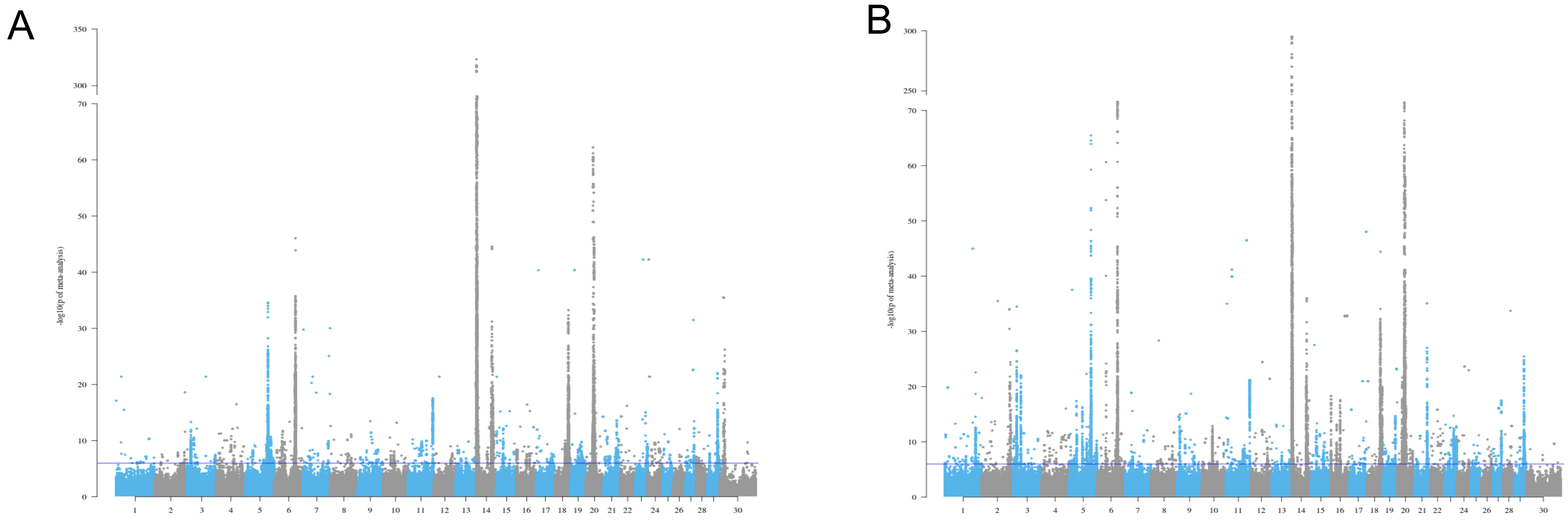

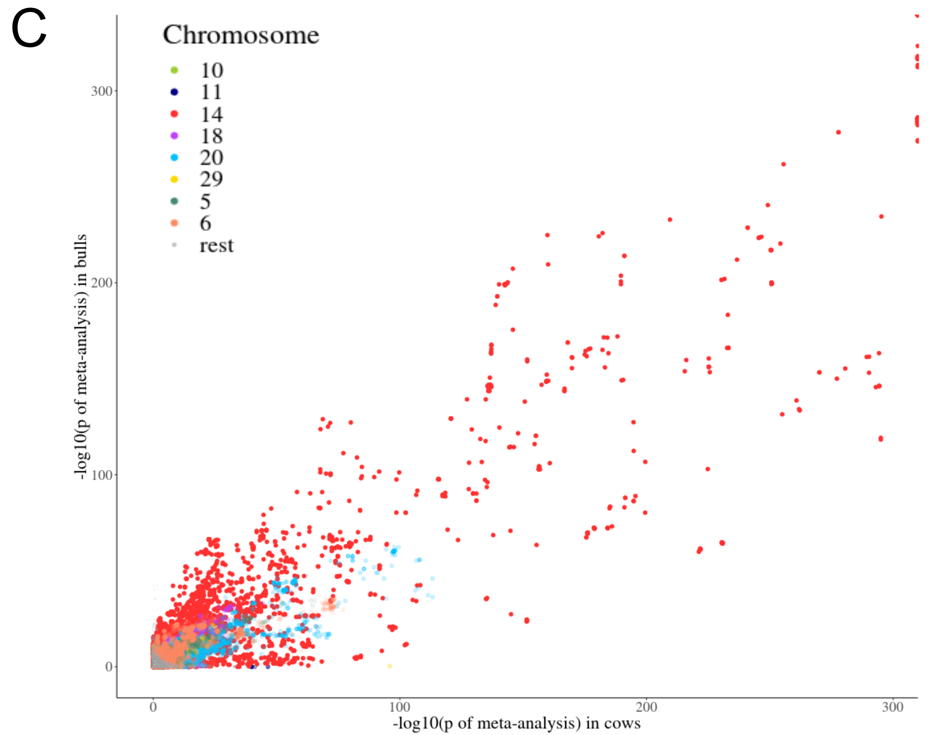

Supplementary Figure 4. The Manhattan plots meta-analysis of genome-wide association studies of 34 traits in bulls (A) and cows (B). C: the scatter plot of p-values of meta-analysis of bulls (Y axis) and of cows (X axis) the dots are variants coloured based on their chromosomes.

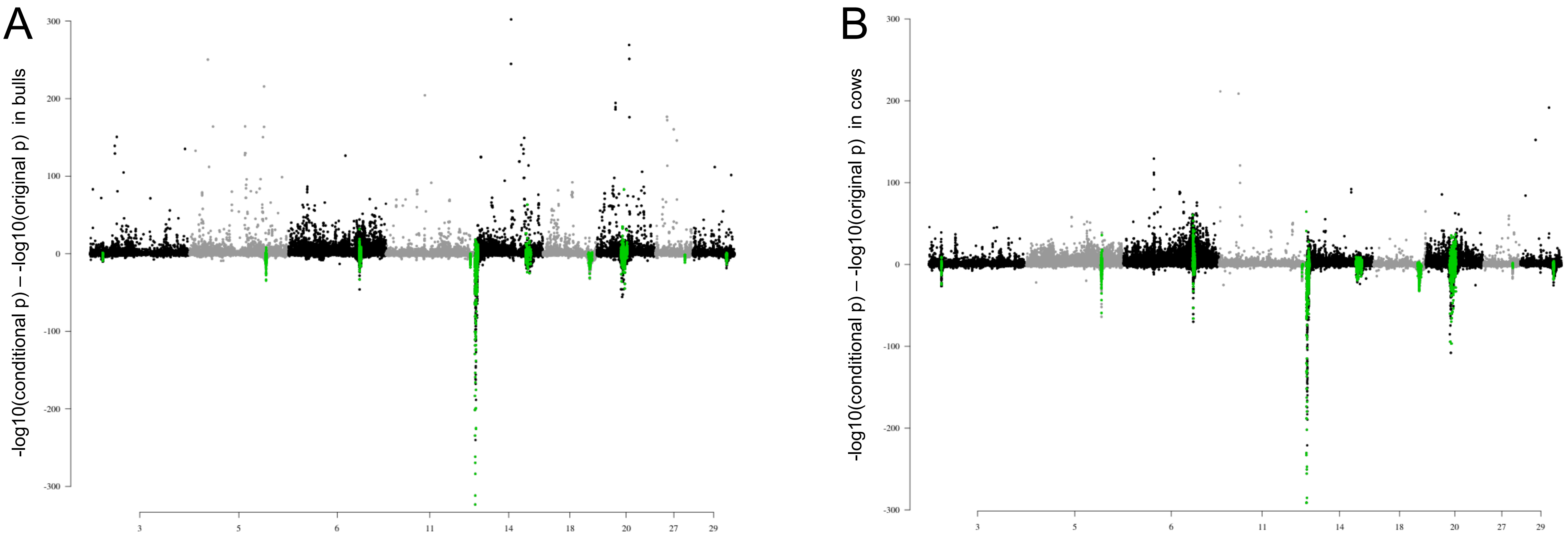

Supplementary Figure 5. Manhattan plots of the p value difference between the multi-trait meta-analysis of conditional GWAS and of original GWAS in bulls (A) and cows (B). The conditional GWAS of 34 traits were performed by fitting the top 100 variants selected from the variants prioritized by the sliding-window clustering. The X-axis was the chromosomes from which the top 100 variants were selected. The y-axis was p difference shown in log10 scale (multi-trait p of conditional GWAS – multi-trait p of original GWAS). Green dots were those variants from the clusters where the top 100 variants were selected.

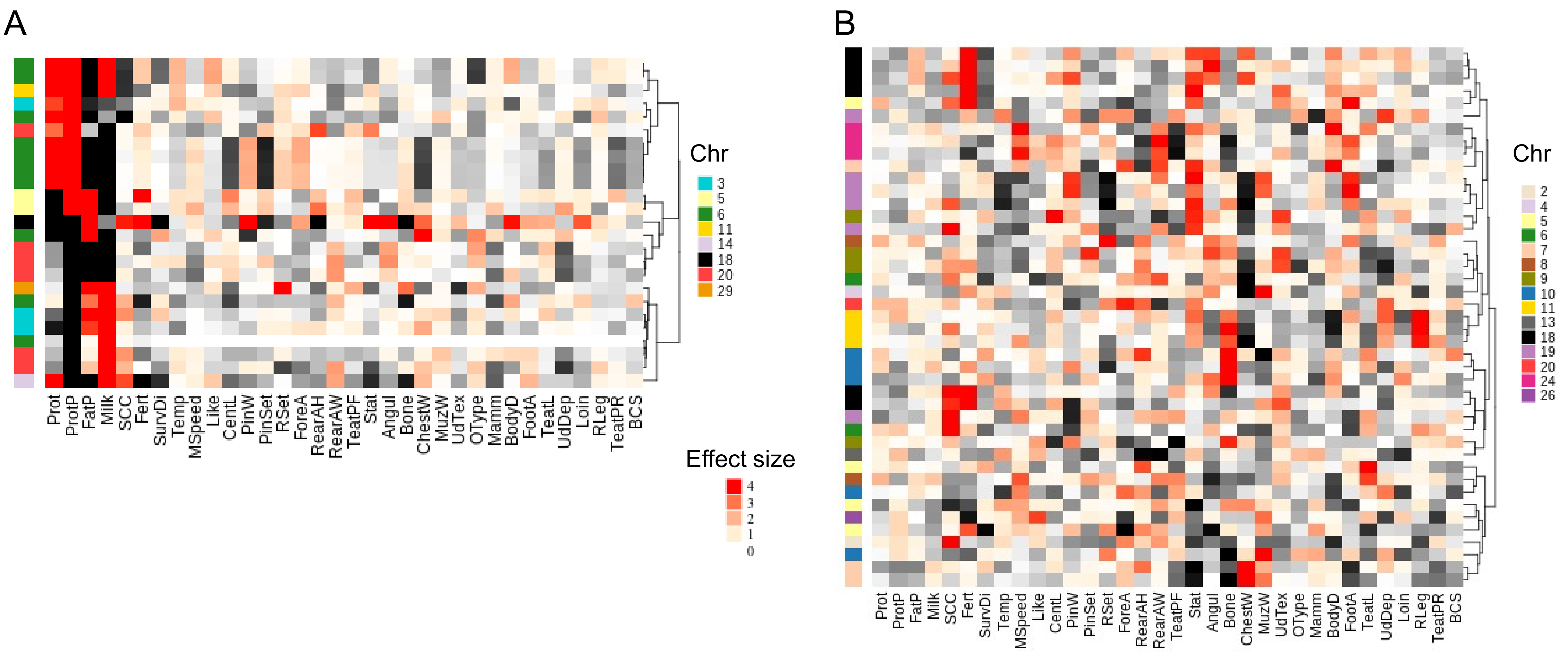

Supplementary Figure 6. Conventional hierarchical clustering of effect size of selected variants across 34 complex traits. **A**: The heatmap for variants with uneven pleiotropic effects, defined as the averaged size effect variations across 34 traits > mean + 3 × standard deviation of mean variation. The clustering was based on the absolute weighted t value (b/se) of bulls and cows. **B**: The heatmap for variants with pleiotropic effects but the pleiotropy was not led by milk production traits (the effect size on protein (Prot), protein percentage (ProtP), fat percentage (FatP) and milk yield (Milk) <2).

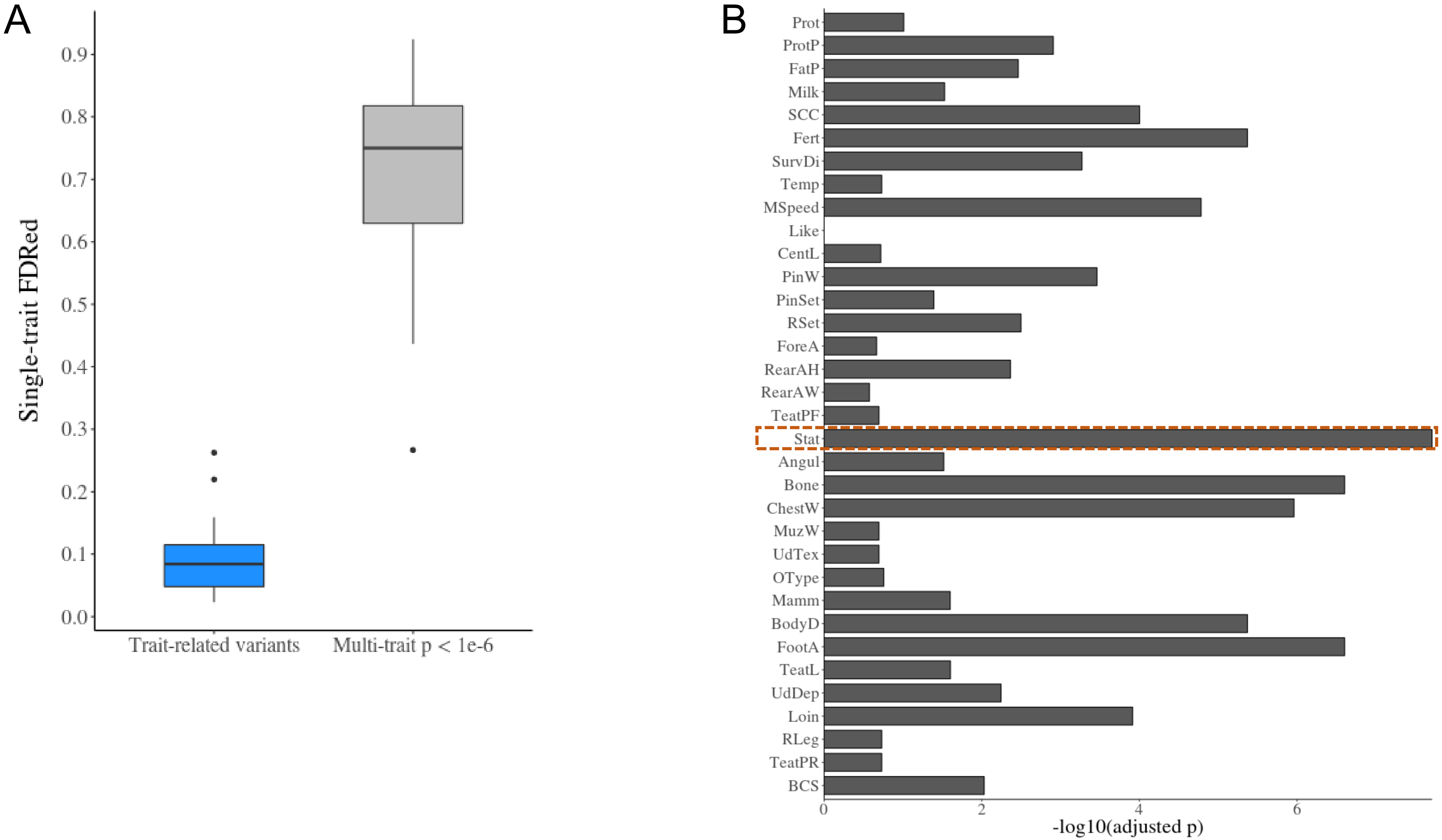

Supplementary Figure 7. Characteristics of trait-related variants. **A**: The single-trait false discovery rate by effect direction (FDRed) calculated using variants identified as related to each trait ($variants_{trait}$) compared with the single-trait FDRed calculated using variants selected by weighted multi-trait p value (p<1e-6, related to any one of the 34 traits). Each boxplot represented the summary stats of the single-trait FDRed for 34 traits. **B**: Enrichment of cattle stature-variants [1] in each set of the dairy cattle trait-related variants. The red bar highlights the strongest enrichment of the cattle stature-variants in the trait stature (Stat) in the current analysis.

[1] Bouwman AC, et al. (2018) Meta-analysis of genome-wide association studies for cattle stature identifies common genes that regulate body size in mammals. Nature genetics 50(3):362.

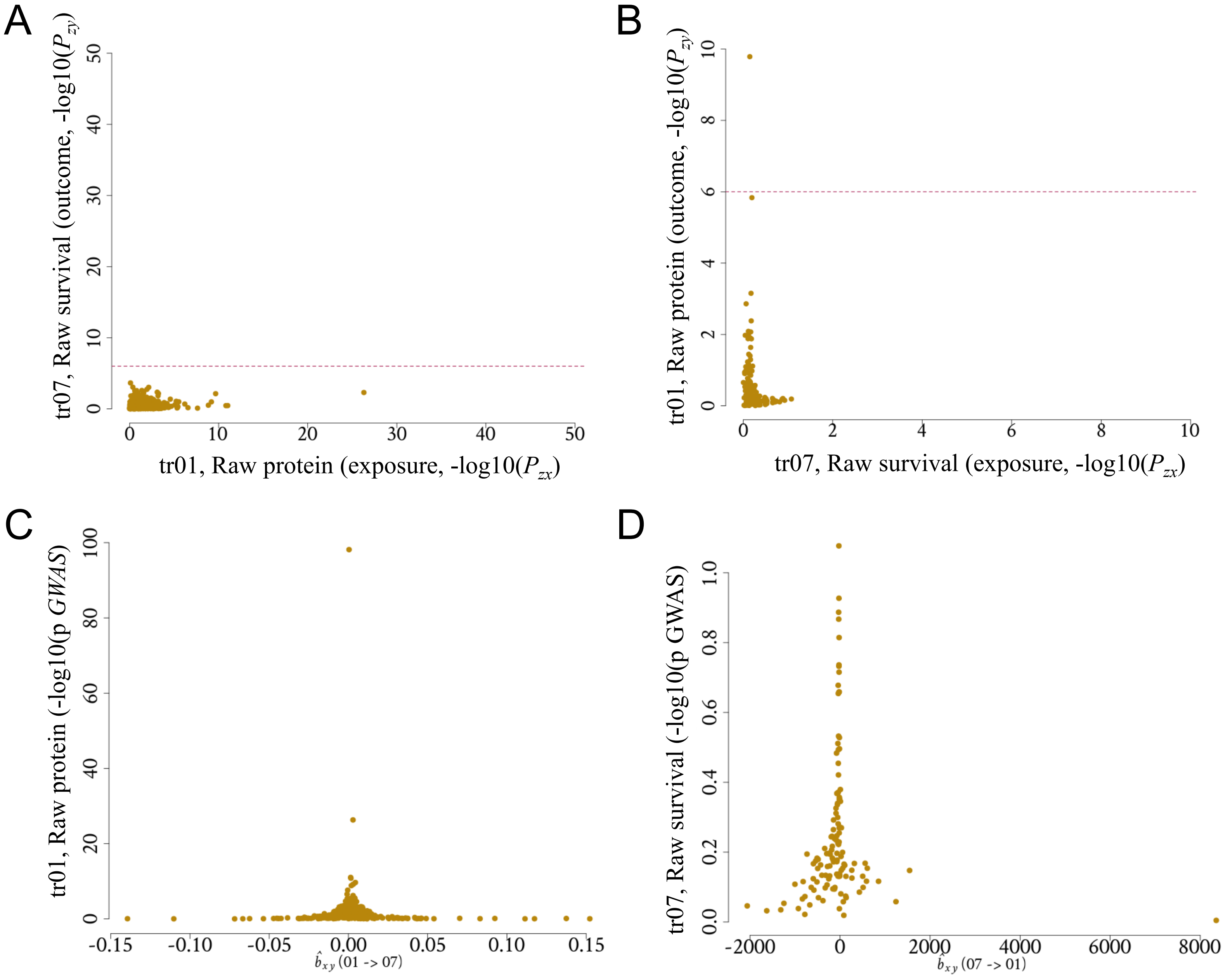

Supplementary Figure 8. Additional plots of the Mendelian Randomisation (MR) analysis between raw trait protein (trait 01) and survival (trait 07). **A**: Association p-values of the genetic instruments from GWAS for raw protein vs. those for raw survival. **B**: Association p-values of the genetic instruments from GWAS for raw survival vs. those for raw protein. The redline indicates the p-value=1e-6. According to [1], points above the redline in plot A and B indicate the existence of pleiotropic outliers in the MR, suggesting the inaccuracy of MR. Here the pleiotropic outliers are observed for the MR of survival causing protein, but not for the MR of protein causing survival. **C**: Funnel plot of b_{xy} vs. GWAS P-value of raw protein. **D**: Funnel plot of b_{xy} vs. GWAS P-value of raw survival. According to [2], the status of asymmetry in funnel plots C and D indicates the existence of directional pleiotropy. Here the directional pleiotropy is likely to exist in the MR of survival causing protein, not in the MR of protein causing survival.

[1] Zhu, Z. et al. Causal associations between risk factors and common diseases inferred from GWAS summary data. Nature communications 9, 224 (2018).
[2] Bowden, J., Davey Smith, G. & Burgess, S. Mendelian randomization with invalid instruments: effect estimation and bias detection through Egger regression. International journal of epidemiology 44, 512-525 (2015).

Supplementary Note 1. Enrichment of functional signals in the single-trait and multi-trait GWAS hits with small effects.

As shown in Figure 2 in the main text, when imposing the same p-value thresholds in the two populations (bulls and cows in this study) for selecting variants, significant differences between the false discovery by effect direction (FDRed) and the conventional FDR were observed. Such differences also existed at relatively lenient p-value thresholds (e.g., p>1e-6). For example, as described in Results, at the p = 1e-3 level, the single-trait FDRed was 0.032(±0.015) while the conventional FDR was 0.98(±0.015) and the multi-trait FDRed was 0.003 while the conventional FDR was 0.49. To test whether variants selected by lenient p-value thresholds in both sexes (which have small FDRed) are informative, we checked the enrichment of conserved sites and expression QTLs [1, 2] in GWAS hits at a set of p-value thresholds from lenient to stringent imposed in bulls and cows (Supplementary Note 1-Table 1).

Supplementary Note 2-Table 1. The number of significant variants (N.variants) at the given p-value thresholds (p) of single-trait and multi-trait GWAS impo sed in both populations (bulls and cows) and the count of the number of these significant variants being conserved sites across 100 vertebrae species (N.conserved), being splicing quantitative trait loci (N.sQTL) and being gene expression QTL (N.geQTL). On the ride side of each of the column of the count of functional variants (N.conserved, sQTL and N.geQTL), the p-values of the significance of the overlap are shown. The functional datasets are obtained from [1, 2]. For single-trait analysis, the counts and p-values are show as the average and the standard error (numbers in parenthesis) for 34 traits.

Type	p	N.variants	-log10 (FDRed)	-log10 (conventional FDR)	N.conserved	-log10($p_{overlap}$)	N.sQTL	-log10($p_{overlap}$)	N.geQTL	-log10($p_{overlap}$)
Single-trait	1	17259277(34306)	0.0(0.0)	0.0(0.0)	367770(898)	0.0(0.0)	984636(3)	0.0(0.0)	93629(0)	0.0(0.0)
	0.5	4251939(24153)	0.1(0.0)	0.0(0.0)	90436(497)	0.8(0.4)	265991(1946)	28.7(11.7)	25946(286)	5.4(2.1)
	0.1	241658(6071)	0.4(0.0)	0.0(0.0)	5287(141)	2.2(0.5)	18296(877)	129.1(20.7)	1936(127)	55.4(13.9)
	5E-02	83965(4088)	0.6(0.0)	0.0(0.0)	1848(91)	1.7(0.4)	6948(602)	110.4(21.0)	775(83)	48.5(13.0)
	5E-04	2333(1065)	214.7(25.6)	0.0(0.0)	46(22)	0.4(0.1)	362(177)	42.9(16.8)	57(24)	20.9(9.3)
	5E-06	990(523)	264.3(20.9)	0.3(0.1)	23(12)	0.4(0.2)	191(104)	29.7(13.6)	40(18)	20.9(9.4)
	5E-08	559(287)	253.9(24.0)	0.5(0.2)	13(7)	0.1(0.1)	123(61)	31.2(14.6)	31(14)	19.9(9.1)
	5E-10	383(201)	316.5(0.0)	0.8(0.3)	10(5)	0.3(0.1)	93(49)	26.5(13.6)	25(12)	18.0(9.1)
Multi-trait	1	17495271	0.4	0.0	373943	0	984644	0.0	93631	0.0
	0.5	5100124	0.6	0.0	111929	22	391290	300.0	40562	300.0
	0.1	641399	1.0	0.0	14646	15	64554	300.0	7079	300.0
	5E-02	307008	1.1	0.0	7026	8	34455	300.0	4002	300.0
	5E-04	31366	2.8	0.6	613	0	5460	300.0	726	187.4
	5E-06	16938	300.0	2.3	293	0	3187	300.0	465	146.8
	5E-08	11987	300.0	4.1	198	0	2249	300.0	384	142.1
	5E-10	9236	300.0	6.0	157	0	1759	300.0	348	149.5

The significance of the enrichment was based on the hypergeometric test in phyper in R (V3.6.1) [3] using four types of count: 1) the number of variants that are both GWAS hits and functional (e.g., variants under conserved sites), 2) the total number of functional variants (e.g., number of variants under conserved sites), 3) the total number of GWAS hits at a given p-value threshold imposed in two sexes (e.g., p=0.01 in GWAS of bulls and cows) and 4) the total number of variants entering the analysis (i.e., 17 million variants). We observed significant enrichment of functional variants, including conserved sites, splicing sQTLs and gene expression geQTLs, at both lenient (e.g., p=5e-02 or 5e-04) and stringent (e.g., p=5e-08 or 5e-10) p-value thresholds. At stringent p-value thresholds, both the FDRed and the conventional FDR were small. However, at lenient p-value thresholds which have significant enrichments of functional variants, the FDRed was much smaller than the conventional FDR.

References:

1. Xiang R, Hayes BJ, Vander Jagt CJ, MacLeod IM, Khansefid M, Bowman PJ, et al. Genome variants associated with RNA splicing variations in bovine are extensively shared between tissues. BMC Genomics. 2018;19(1):521. doi: 10.1186/s12864-018-4902-8.
2. Xiang R, Berg Ivd, MacLeod IM, Hayes BJ, Prowse-Wilkins CP, Wang M, et al. Quantifying the contribution of sequence variants with regulatory and evolutionary significance to 34 bovine complex traits. Proceedings of the National Academy of Sciences. 2019;116(39):19398-408. doi: 10.1073/pnas.1904159116.
3. Team RC. R: A language and environment for statistical computing. . Vienna, Austria.: R Foundation for Statistical Computing; 2017.
4. Xiang R, MacLeod IM, Bolormaa S, Goddard ME. Genome-wide comparative analyses of correlated and uncorrelated phenotypes identify major pleiotropic variants in dairy cattle. Scientific Reports. 2017;7(1):9248.
5. MacLeod I, Bowman P, Vander Jagt C, Haile-Mariam M, Kemper K, Chamberlain A, et al. Exploiting biological priors and sequence variants enhances QTL discovery and genomic prediction of complex traits. BMC genomics. 2016;17(1):144.
6. Kemper KE, Reich CM, Bowman PJ, Vander Jagt CJ, Chamberlain AJ, Mason BA, et al. Improved precision of QTL mapping using a nonlinear Bayesian method in a multi-breed population leads to greater accuracy of across-breed genomic predictions. Genetics Selection Evolution. 2015;47(1):29.

7. Littlejohn MD, Tiplady K, Fink TA, Lehnert K, Lopdell T, Johnson T, et al. Sequence-based Association Analysis Reveals an MGST1 eQTL with Pleiotropic Effects on Bovine Milk Composition. Scientific Reports. 2016;6:25376. doi: 10.1038/srep25376

https://www.nature.com/articles/srep25376#supplementary-information.

8. Pausch H, Emmerling R, Gredler-Grandl B, Fries R, Daetwyler HD, Goddard ME. Meta-analysis of sequence-based association studies across three cattle breeds reveals 25 QTL for fat and protein percentages in milk at nucleotide resolution. BMC genomics. 2017;18(1):853.

Supplementary Note S2. Notable variants/loci associated with multiple CT traits.

There were over 2,000 variants with uneven pleiotropic effects, defined as those variants with a large trait-wise variance of effect size (mean of variance of effect size across 34 traits > 3 × standard deviation, Supplementary Data 7). The conventional hierarchical cluster analysis of these variants revealed that their pleiotropy was dominated by their large effects on the milk production CT traits, including fat percentage (FatP), milk yield (Milk), protein percentage (ProtP) and protein yield (Prot) (Supplementary Figure 6a). Most of these variants tagged or were very close to previously reported large QTL in dairy cattle (Supplementary Data 7). The conventional hierarchical cluster analysis of those uneven pleiotropic variants also revealed some patterns among the effects (Supplementary Figure 6a). For example, variants from chromosome 6 can also be grouped based on whether they only affected protein yield and/or CT fat percentage (>Chr6:87.1Mb, tagging *CSN1S2* and *CSN3*) or affected both CT milk production traits and some CT type traits (<=Chr6:87.1Mb, tagging *ABCG2* and *SULT1D1*).

To better understand pleiotropic effects not dominated by production traits, variants with $|t| < 2$ on the CT milk production traits and within the top 1000 ranking for their Π values were selected and further analysed (Supplementary Data 8). These variants had the maximum p_{wm} = 2.4e-07 and the conventional hierarchical cluster analysis of an LD pruned set of them (LD $r^2 < 0.1$ in 2Mb sliding-windows) showed that they were spread across chromosomes and traits (Supplementary Figure 6b). These variants included ones close to previously discovered SNPs from the cattle *CTU1* loci (Chr18:57.6Mb) that associated with milk production traits, fertility (fert) and stature (stat) [4]. However, there were also novel trait related mutations identified, including variants with effects on CT fertility and somatic cell count (SCC) tagging *ZNF331* (ENSBTAG00000016513, Chr18:61M), variants with effects on CT bone

quality (Bone) and teat length (TeatL) tagging the homeobox gene *MEIS2* (Chr10:32.7M), variants with effects on CT stature, rear legs set (RSet) and many other type traits tagging *STX8* (Chr19:29.4M) and variants with effects on CT body depth (BodyD) and rear leg view (RLeg) tagging *ADCY3* (Chr11:74.5M). In addition, variants from different chromosomes tagging *WSCD1* (Chr19:26.2M) and *RPLP2* (ENSBTAG00000003086, Chr7:33.6M) and close to *RAI14* (Chr20:39529782) showed pleiotropic effects on CT udder depth (UdDep), rear attachment height (RearAH) and fore attachment (ForeA) (Supplementary Figure 6b and Supplementary Data 8).

Pleiotropic variants with unique effect patterns across CT traits identified in this study are novel to the genetics of cattle complex traits. Because the commonly known major loci of dairy cattle such as *DGAT1*, *CSN*, *MGST1*, and *PAEP* have dominating effects on a large number of traits (see [4-8]), it might be assumed that that most of the pleiotropic effects in dairy cattle are driven by the major milk production trait loci. While we show that many pleiotropic variants were related to these major dairy production QTL, a sizable number of variants identified in the current study showed pleiotropic effects led by the non-milk production traits such as CT fertility and survival, teat length and udder depth (Supplementary Data 8 and Figure 6). The identification of those variants with lead pleiotropic effects on non-production traits can be used for customising SNP arrays for the dairy cattle breeding industry.
